# Aerosol Nutrients and Their Biological Influence on the Northwest Pacific Ocean (NWPO) and Its Marginal Seas

**DOI:** 10.3390/biology11060842

**Published:** 2022-05-30

**Authors:** Cui Guo, Yao Zhou, Hongyan Zhou, Chang Su, Liangliang Kong

**Affiliations:** 1 College of Marine Life Sciences, Ocean University of China, Qingdao 266003, China; areadyzhou@163.com (Y.Z.); zhy6556@stu.ouc.edu.cn (H.Z.); suchang6563@stu.ouc.edu.cn (C.S.); 2 Institute of Evolution and Marine Biodiversity, Frontiers Science Center for Deep Ocean Multispheres and Earth System, Ocean University of China, Qingdao 266003, China; 3 Department of Biology, McGill University, Montréal, QC H3A 1B1, Canada

**Keywords:** atmospheric deposition, East Asia, northwest Pacific Ocean, marine phytoplankton, marine bacteria

## Abstract

**Simple Summary:**

With intensifying human activities in the past decades, East Asia has recorded increasingly severe air pollution and become the second largest aerosol source on earth. The large quantity of aerosol emissions is not only a major health threat to humans, but can also be transported for a long distance and deposited in downwind seas and oceans. The aerosol contains major ions, heavy metals, and organic matters that are important external nutrients in upper oceans and potentially influence marine microbes and biogeochemical cycles. Therefore, the role of atmospheric deposition to oceans has received growing attention in recent years. In this paper, the current state of knowledge on the atmospheric nutrients and the biological effect of East Asian aerosol deposition on the northwest Pacific Ocean are reviewed, which could help us better understand the comprehensive influence of East Asian aerosols on marine ecosystems, and give insights into future research directions, especially under the future scenarios of changing human activities and climate.

**Abstract:**

Atmospheric deposition is recognized as a significant source of nutrients in the surface ocean. The East Asia region is among the largest sources of aerosol emissions in the world, due to its large industrial, agricultural, and energy production. Thus, East Asian aerosols contain a large proportion of anthropogenic particles that are characterized by small size, complex composition, and high nutrient dissolution, resulting in important influences on marine microbes and biogeochemical cycles in the downwind areas of the northwest Pacific Ocean (NWPO). By using remote sensing, modeling, and incubation experimental methods, enhanced primary production due to the East Asian aerosol input has been observed in the NWPO, with subsequent promotion and inhibition impacts on different phytoplankton taxa. Changes of bacterial activity and diversity also occur in response to aerosol input. The impact of East Asian aerosol loadings is closely related to the amount and composition of the aerosol deposition as well as the hydrological condition of the receiving seawater. Here, we review the current state of knowledge on the atmospheric nutrients and the effects of the East Asian aerosols on microbes in the NWPO region. Future research perspectives are also proposed.

## 1. Introduction

**Figure 1:**
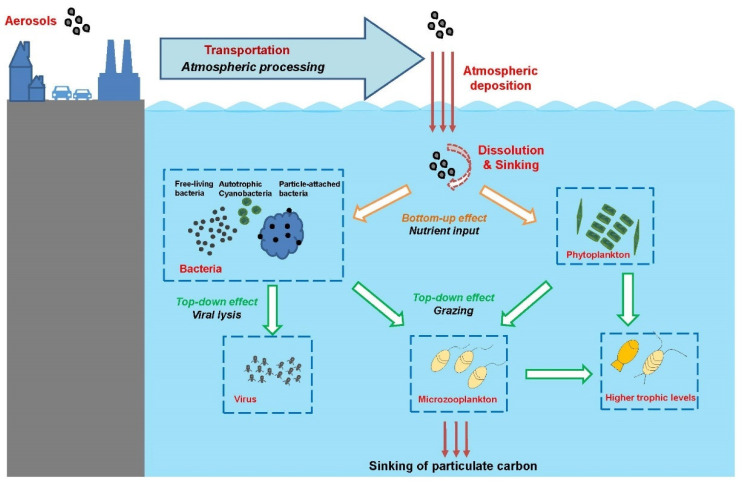
Impacts of aerosol deposition on the marine food web. Aerosols from natural and anthropogenic sources are transported to ocean regions. The solubility of nutrients in the aerosols can be enhanced by atmospheric processes such as acidification, photochemical, or cloud processes before being deposited to the seawater. In the ocean, the dissolved aerosol nutrients can be cycled through microbial food web, influencing microbial metabolism, community composition, and biogeochemical cycles, and/or sink in the deep ocean.

Atmospheric particulate matter (also known as aerosols) is microscopic particles of solid or liquid matter suspended in the air. These particles are transported from the atmosphere to terrestrial and aquatic ecosystems via a process referred to as atmospheric deposition. Since industrialization, intensifying human activities have created increasing emissions of atmospheric aerosols. It is estimated that about 420–480 Tg of aerosols are transported and deposited into the ocean every year globally [1,2]. Through this important process, substances in the aerosols, including inorganic nutrients, metal elements and organic pollutants, are transported to seas and oceans [3]. Atmospheric deposition has been shown to be one of the major external sources of nutrients in the ocean and has an important impact on marine microbial food webs and global biogeochemical cycles (Figure 1) [4,5,6]. Thus, determining how atmospheric deposition affects marine ecosystems has become a key and urgent topic in the field of oceanography. In the SOLAS program (Surface Ocean–Lower Atmosphere Study) research plan from 2015 to 2025, “atmospheric deposition and marine biogeochemistry” have been listed as one of five core themes, focusing on the response of marine biogeochemical and biological processes to atmospheric deposition from anthropogenic and natural sources. International research programs such as GEOTRACES (an international study of marine biogeochemical cycles of trace elements and their isotopes) and IMBeR (Integrated Marine Biosphere Research) also include the impact of atmospheric deposition on the ocean as an important topic.

## 2. Global Patterns of Atmospheric Input to the Ocean

The distribution of atmospheric deposition has large geographical variability, which should be considered when predicting its potential impact on marine ecosystems. Attempts have been made to describe the patterns of major aerosol and/or dust sources, which are concentrated in arid or polluted regions of North Africa, the Middle East, and East Asia. These regions form a ‘dust belt’ extending from the west coast of North Africa eastward to the Pacific coast of China [7], corresponding to areas of high atmospheric deposition over the North Atlantic and Mediterranean Sea, which are exposed to the north African desert, China’s marginal seas and NWPO, which are downwind of East Asia [3,8,9,10,11,12,13]. Other marine ecosystems, such as the northern Indian Ocean, are affected by Central Asia and the Indian desert [14]. The Southern Ocean, with a high-nutrient, low-chlorophyll (HNLC) condition, is affected by the Australian desert [15]. Dust sources in the northern hemisphere are far more active and larger than those in the southern hemisphere [7]. Among the major oceans, the North Atlantic receives the highest amount of dust deposition, 178–259 Tg year^−1^; followed by the Indian Ocean and North Pacific that receive dust deposition of 29–154 and 31–96 Tg year^−1^, respectively; the South Pacific and South Atlantic only receive dust deposition of 8–29 and 5-35 Tg year^−1^, respectively [16,17,18,19,20].

## 3. Features of East Asian Aerosols

The East Asia region has undergone rapid urbanization, industrial growth, and increasing energy consumption over the past few decades. Inevitably, deteriorating air quality has accompanied these processes [21,22], making East Asia the second largest global aerosol source after the Sahara. Approximately 31–96 Tg of East Asian dust and aerosols are being continuously transported and deposited into the North Pacific Ocean every year, accounting for 10–25% of total global dust emissions [7]. Gao et al. [23] reported that the atmospheric deposition of dust into the Chinese marginal seas is 67 Tg yr^−1^, accounting for 14% of the total atmospheric dust deposition to the entire North Pacific. Thus, their potential impacts on the marine ecosystem have been receiving growing attention. 

Compared with the mineral dust from the Sahara Desert (the world’s largest source of dust), the composition of East Asian aerosol is more complex, including not only the mineral dust from the Gobi and Taklimakan deserts, but also a significant amount of anthropogenic aerosols from industrial and agricultural emissions [24,25]. Since the East Asian aerosols are largely associated with haze pollution, they have higher contents of combustion-derived nitrogen and sulfur, heavy metals such as iron, copper, and lead, and complex organic compounds [6,26]. The atmospheric inputs of nitrogen and trace metals from East Asian aerosols to the NWPO are among the highest in the world [8,9,27,28]. 

The particle size of aerosols varies according to their source. Mineral dust is relatively coarse while pollution and smoke particles are much finer [11]. The smaller particle sizes allow transport by monsoonal winds over a much greater distance, and these can then be deposited in nearby seas and oceans [2,29]. It has been reported that fine particles dominate the size spectrum over the Northern South China Sea (SCS) [30]. Using data from satellite sensors, Lin et al. [11] demonstrated that the SCS receives anthropogenic aerosols mixed with dust during dust outbreaks from November to April, when the northeast monsoon prevails, while smoke particles associated with biomass burning in Borneo and Sumatra, from June to September, predominate during the southwest monsoon. The spatial and temporal variability of aerosol sources adds to the complexity of atmospheric impact on the biogeochemistry of the NWPO. 

## 4. Key Species of Atmospheric Nutrients

### 4.1. Atmospheric Nitrogen (N) Input to the Ocean

Most atmospheric N deposition exists in the form of inorganic N, including both oxidized and reduced forms. The oxidized species principally include aerosol nitrate (NO_3_^-^) and gas phase oxides of nitrogen (NO, NO_2_, HNO_3_ and related species). Combustion of fossil fuels is the major anthropogenic source of NO_x_. The reduced N species are found in the form of aerosol NH_4_ and gaseous NH_3_ [8,31,32]. Most NH_y_ is emitted directly from soils or other agricultural sources such as fertilizer and breakdown of urea from domestic animals. Organic nitrogen (ON) is also an important fraction of atmospheric N, comprising ~30% of total N emissions [4,5,33,34]. In a global model-based analysis, the annual flux of inorganic nitrogen emissions is 90.7 Tg N yr^−1^, of which 69% is in gaseous form and 31% in particle form; while ON emission is estimated to be 31.6 Tg N yr^−1^ and 30% is in gaseous form and 70% in particle form [5]. ON in rainwater and aerosols is composed of a variety of compounds with diverse origins. High-molecular-weight ON (>1000 Da) and low-molecular-weight ON (<1000 Da) have been suggested to comprise 57% and 43% of the dry deposited DON (Chen et al. 2010), and 16% and 84% of the wet deposited DON in the East China Sea (ECS) [35]. 

A deficit in the bioavailable N supply required to support new production characterizes large segments of the world’s oceans [4,36]. It has been reported that as much as ~70% of oceanic surface waters (including areas that are co-limited by other elements) are potentially seasonally N limited [4]. Atmospheric N deposition has become one of the main sources of external N for the ocean. Seawater N can be supplied by riverine input, N fixation, and atmospheric deposition. However, the effect of riverine N input (~50–80 Tg year^−1^) on open oceans can be ignored since it is largely confined to coastal and estuarial regions [37,38]. Now, in many estuarine and coastal zones, the atmospheric N loading could equal or even exceed riverine N inputs because of the dramatic increase in anthropogenic emissions [31]. Globally, atmospheric deposition could bring ~39–67 Tg N year^−1^ to the ocean [39], which is ~3 fold higher than in the preindustrial period, and is predicted to grow over the coming decades [4,40,41]. The quantity of atmospheric N deposition is also approaching that of global oceanic N_2_ fixation (~100 Tg year^−1^) [42,43,44,45], and could account for up to about a third of the ocean’s total external N supply [4]. Increase of atmospheric N deposition in the absence of adequate P deposition could result in P limitation of N_2_ fixation in the surface layer, which could lead to decreased competitiveness of diazotrophs and thus a decrease in N_2_ fixation [46,47]. However, the influence of atmospheric N deposition on the ambient seawater N concentration is too small to directly inhibit nitrogenase activity and suppress N_2_ fixation [4]. As such, the supply ratio of N:P:Fe from atmospheric deposition, and whether N_2_ fixation is limited by P or Fe, influence the long-term effect of N deposition on N_2_ fixation. In the SCS, the atmospheric deposition of N has exceeded N_2_ fixation and riverine input, especially in the ocean basin, contributing ~20% of the new production in the oligotrophic waters of the SCS [48,49]. In the marginal seas of NWPO, the increase in N availability has been shown to be mainly driven by increasing deposition of pollutant N from atmospheric sources [10], and NO_3_^−^ and NH_4_^+^ in airborne particles and rainwaters could contribute to ~1.9% of the primary production in the East Asian marginal seas [50]. Under future scenarios of increasing global warming and ocean stratification, the external supply of atmospheric N to the ocean is expected to be more important to primary production and the N cycle in the N-limited NWPO.

### 4.2. Atmospheric Fe and Other Trace Metals Deposition

Fe is an essential micronutrient required in a variety of enzyme systems that are responsible for many important biological processes, such as photosynthesis, respiration and N fixation. Areas affected by Fe limitation have been found to cover ~30% of the world ocean, especially in the HNLC regions of the subarctic North Pacific, the equatorial Pacific and the Southern Ocean [51,52,53,54]. Large expanses of low-nutrient low-chlorophyll (LNLC) waters such as in the tropical South Pacific gyre are also Fe limited [55]. Every year, an estimated flux of 16–32 Tg Fe enters the ocean by atmospheric deposition [1,9], recognized as a major transport path and the dominant source of new dissolved Fe entering open ocean, although the regional situation is more complex. Atmospheric aerosols can be an important source of other bioactive trace metals, such as Zn, Co, Ni, Mn, and Cu [8], which are also required as co-factors for many important molecules involved in marine phytoplankton and bacteria physiology and function. The evidence for significant widespread co-limitation of marine phytoplankton or bacterial growth by many of these trace metal elements, apart from Fe, remains equivocal [56]. 

It is important to consider the solubility of these aerosol metals when assessing their biological impacts on marine microorganisms. The solubility is driven by variability in sources, atmospheric processing, and the physical, chemical, and biological features of the receiving seawater. For example, mixing of dust with acid gases is likely to enhance the solubility of the Fe during long-distance transport in the atmosphere [57]. Thus, although the emissions of combustion-derived Fe and other metals are much lower than mineral dust metals, they supply more bioavailable metals than the mineral dust and are especially important in high anthropogenic emission regions [58,59]. The speciation and bioavailability of some biologically important metals in seawater, i.e., Fe, Cu, and Co, are largely controlled by biogenic ligands [60]. The dissolution process can be either rapid or gradual, i.e., Zn, Co, and Cd dissolve faster than other metals, and Ni, Cu, and Mn dissolve slower [59]. Of global emission sources, Asia provides the largest quantity of anthropogenic trace metals [28]. However, constrained by very limited observations, there are still large uncertainties in our understandings of the availability and impacts of atmospheric metal deposition on marine microbes. 

### 4.3. Atmospheric Phosphorus (P) Deposition

Compared with the N and Fe supply, the atmospheric supply of P is much lower, resulting in a much higher N:P ratio than the Redfield ratio (N:P = 16:1), deemed most suitable for optimal marine phytoplankton growth [6,61,62,63]. Atmospheric P deposition, primarily associated with lithogenic dust, is less perturbed by human activity. In East Asian aerosols dominated by human sources, the N:P ratio can reach >500 [62,64], whereas in the P-limited Mediterranean Sea, Sahara dust input might relieve P limitation and stimulate phytoplankton growth [13,65]. The annual emission of atmospheric P in fine and coarse particles (<10 μm) is approximately 1 Tg, with only 0.25 Tg thought to be soluble [61]. Enhancement of P solubility may occur due to acidification during long-range transportation [66]. It has been observed that P in East Asian anthropogenic particles has a higher solubility than the mineral dust, due to the acidification processes associated with the formation of sulfate and nitrate, suggesting that the air pollution in East Asia might have elevated the input of bioavailable P to the downwind seas [67].

### 4.4. Atmospheric Deposition of Oorganic Matters

In the past, inorganic nutrients and metals in aerosols were thought to be the main factors affecting marine ecosystems and few studies focused on the biological effects of organic matter in aerosols. In fact, organic matter is now known to comprise a considerable proportion of the aerosols, especially in East Asia where combustion of fossil fuel and biomass results in the emission of large amounts of black carbon and organic compounds [5,26,68]. The proportion of organic compounds in East Asian aerosols can reach 30–90%, of which about 16–50% are water soluble [69,70]. This soluble organic matter contains both refractory organic carbon and labile organic carbon, the latter of which may also exert a role in regulating the growth of marine bacteria. It has been reported that the labile portion of organic compounds can be efficiently taken up by bacteria [71], while other studies have noted the recalcitrant character of the fluorescent dissolved organic matter associated with atmospheric aerosols [72]. However, study of the effect and bioavailability of atmospheric organic matter is still in its infancy. How atmospheric organic matter input influences bacterial growth and diversity, metabolic function, the microbial carbon pump, and the carbon cycle in the marine ecosystem is still not clear.

## 5. Effect of East Asian Aerosol on Phytoplankton in NWPO and Its Marginal Seas

### 5.1. Fertilizing Effect

As an important source of nutrients to the ocean, atmospheric deposition has been recognized to exert an important role in regulating primary production and phytoplankton growth (Figure 1). Assessments based on modeling and remote sensing data suggest that the aerosol input has a close connection with biological production and coastal eutrophication. By analyzing more than 10 years of satellite records of Asian dust events and remotely sensed chlorophyll *a* concentrations, many studies have identified significant correlations between chlorophyll *a* concentrations and aerosol optical depth, a proxy for atmospheric dust input and nutrient supply, in NWPO and the adjacent China Seas [49,73,74,75,76,77]. Strong dust events could enhance phytoplankton biomass by more than 2-fold (estimated by increase in chlorophyll *a* concentrations) that could account for up to 70% increase in ocean primary production and trigger phytoplankton blooms in the northern SCS and NWPO [49,76,77]. They also found that the stimulation effect of atmospheric aerosol was greater in the central basin where other sources of nutrient inputs (e.g., river runoff or upwelling) were lower [78]. It is suggested that atmospheric Fe input has a fundamental effect on phytoplankton growth in China Seas and could explain 5–68% of the phytoplankton growth [79,80,81]. Atmospheric N deposition can support >10% of the annual export production in nearshore regions along the Japanese coast and the SCS [49,82]. Using sediment trap measurement and a biogeochemical model, it has been shown that the seasonal variability of deep-ocean POC export is largely driven by the atmospheric Fe and N deposition that cause seasonal change of phytoplankton community composition and micro- and meso-zooplankton grazing pressure [83]. In addition to direct stimulation by atmospheric nutrients, strong winds accompanying the dust storms can also induce vertical mixing of the water column and the supply of nutrients into the mixed layer from the subsurface [49,84], the effect of which may occasionally overwhelm the effect of atmospheric input of aerosol nutrients [73]. 

Bottle incubation-based microcosm assays provide solutions to evaluate direct effects of atmospheric deposition of Asian dust on phytoplankton growth and identify specific contributions of aerosol nutrients. Amendment of dust, haze particles or rainwater into seawater samples caused significant increase in chlorophyll *a* concentration by up to 4-fold in NWPO and its marginal Seas (Table 1). The stimulation effect was more profound in oligotrophic than mesotrophic waters [64,85], while aerosol addition had little or no effect on phytoplankton growth in some eutrophic waters [86,87,88]. However, considering the fast nutrient dispersion and high sinking rates of aerosols in the in situ seawater, the effect of aerosols in the real marine environment may be less significant than that in the microcosm experiments.

### 5.2. Stimulation of N_2_ Fixation

Response of marine N_2_ fixation to aerosol deposition is also of particular interest because growth of nitrogen-fixing organisms could be limited by Fe and P in ocean ecosystems [36]. Most studies focus on the effects of aerosol Fe, because N_2_ fixing diazotrophs require a high amount of cellular Fe as an important cofactor of the nitrogenase enzyme that catalyzes N_2_ fixation [89]. Meanwhile, dissolved Fe is present at extremely low concentrations (<0.1 nM) in surface waters of the open ocean [56] that could limit growth of marine phytoplankton including diazotrophs [90,91]. N_2_ fixation rate was significantly enhanced by addition of Saharan mineral dust in the Mediterranean Sea [92,93,94] and North Atlantic [36] and the stimulation effect was attributed to the supply of Fe and P in the dust. The availability of N, P, and Fe and their ratios in the aerosol and ambient seawater could largely determine the trend and the extent to which aerosol addition could influence N_2_ fixation in the ocean. For example, addition of Saharan mineral dust with a lower N:P ratio into the Eastern Mediterranean stimulated N_2_ fixation rates more prominently compared to anthropogenic European aerosols with a higher N:P ratio [94]. A recent study reported supply ratio of Fe:N from subsurface layers is the most important factor in regulating diazotroph abundances and N_2_ fixation rates across the tropical NWPO, while phosphate availability sets an upper limit of total amount of fixed N [95]. As the atmospheric P inputs were strongly depleted relative to N and Fe in the context of the stoichiometry of phytoplankton Fe, N, P requirements, especially in anthropogenic aerosols [96], deposition of anthropogenic East Asian aerosol may fuel diazotrophs with more stoichiometrically available Fe than P in the NWPO region and its marginal seas by providing anthropogenic East Asian aerosols, causing the switch of N_2_ fixation from Fe to P limitation. In the northern SCS receiving East Asian aerosols, co-limitation of N_2_ fixation by both Fe and P have been demonstrated by nutrient addition assays [95]. However, the effect of atmospheric input on N_2_ fixation in NWPO is still unknown.

Growth of marine diazotrophs, particularly the prominent genus *Trichodesmium*, benefit from aerosol additions [93]. *Trichodesmium* can actively acquire nutrients from airborne dust by multiple pathways and strategies, including efficient dust capturing and centering in the colony [97], sensing particle composition and selective collection of nutrient-rich (i.e., Fe-rich, P-rich) particles [98,99], and mutualistic interactions between *Trichodesmium* and associated bacteria for utilization of iron from dust [100]. In addition, the heterotrophic bacterial N_2_ fixers [94] and other N_2_-fixing unicellular cyanobacteria [101] have also been reported to prevail after aerosol addition.

### 5.3. Change of Nutrient Stoichiometry 

A number of bottle incubation experiments have been conducted to demonstrate the detailed response of phytoplankton to atmospheric deposition in NWPO. From these nutrient enrichment experiments, it was found that the addition of inorganic N and aerosols both caused a significant increase in phytoplankton biomass, although the promotion effect of aerosol or dust addition was usually greater when the same amount of inorganic N was added to oligotrophic seawaters [64,87]. This suggests that the East Asian aerosols stimulate phytoplankton growth by supplying not only N but also other components, possibly Fe, in LNLC regions [64]. However, by providing excess N but negligible amounts of P, the atmospheric input may increase the N:P ratio and cause P limitation in the oligotrophic seawater. Thus, adding additional P with aerosols sometimes stimulated a larger increase in chlorophyll *a* concentration than by adding aerosols alone [86], especially in coastal or estuarine regions where the N:P ratio is usually higher. In the oligotrophic seawaters of the SCS and the subtropical gyre of NWPO, a combination of N, P, and Fe addition was observed to have the strongest stimulation effect in multiple nutrient addition experiments [85,87]. 

### 5.4. Shift of Community Composition and Struture

**Figure 2:**
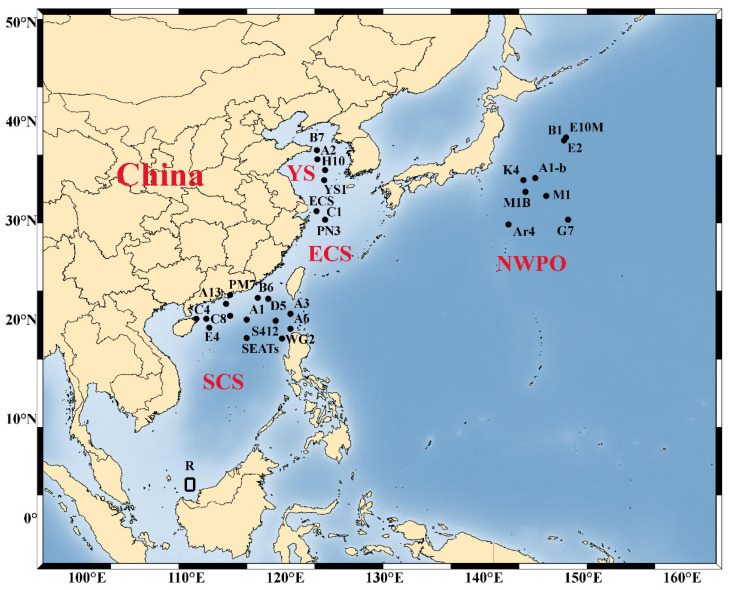
Sampling sites for aerosol amendment bioassays conducted in northwest Pacific Ocean (NWPO) and its marginal seas. ECS, East China Sea; SCS, South China Sea; YS, Yellow Sea.

Alleviation of nutrient limitation and change of nutrient stoichiometry by atmospheric input can further drive changes in phytoplankton community composition and physiological state. Bioassays showing the change of phytoplankton biomass and community structure change with dry and wet deposition amendment in the NWPO and its marginal seas are summarized in Table 1, and the study sites are shown in Figure 2. In general, the larger micro-phytoplankton (20–200 μm) derive more benefit from the input of atmospheric nutrients than pico- (0.2–2 μm) and nano-sized cells (2–20 μm), leading to a shift in size structure of the phytoplankton community [64,87,102,103]. However, the beneficial phytoplankton taxa were not consistent across NWPO, due to differences in nutrient stoichiometry of the experimental sites and different sources of aerosols. After the addition of East Asian aerosols to the oligotrophic SCS and Kuroshio extension region, the phytoplankton community composition shifted to diatoms (N:P < 16), while it shifted to dinoflagellates in the ECS (N:P 16), due to the different nutrient requirement of the two taxa [64,88]. Using the amplicon sequencing of the rbcL gene method, Meng et al. [104] observed different changes in the phytoplankton community structure after adding aerosols from different sources: mineral dust resulted in a significant increase in the relative abundance of *Haptophyceae*, while aerosols with the highest N led to the largest increase in *Bacillariophyceae* (diatoms), *Dinophyceae* (dinoflagellates), and *Cryptophyceae*. Among the diatom species, *Pseudo-nitzschia*, *Nitzschia,* and *Chaetoceros* usually accounted for the largest increases in response to aerosol addition [64,85].

The shift of phytoplankton size and community structure from pico- to nano- and micro-phytoplankton in response to aerosol addition has important biogeochemical implications in the NWPO. For example, in the oligotrophic SCS that is dominated by picophytoplankton, the community composition shift to diatoms may contribute more to vertical carbon export through sinking of senescent cells [64]. Meanwhile, increased phytoplankton biomass and change of community composition can stimulate grazing activities from higher trophic levels and thus enhance the carbon export through downward zooplankton fecal pellets or detritus [64,102]. All these changes can enhance the biological pump and potentially change the carbon budget in the oligotrophic SCS.

### 5.5. Inhibitory Effect

Atmospheric deposition has also been demonstrated to have an inhibitory effect on phytoplankton growth, especially in pico- and nano-phytoplankton. The negative effect was mostly attributed to the toxicity of some trace metals in the aerosols, such as Cu and Cd [27,108]. However, the current understanding of the toxic effects of East Asian atmospheric deposition on phytoplankton is very limited. Metal toxicity has been found across many phytoplankton taxa with different abilities to tolerate toxic metals and different toxicity thresholds [109,110,111]. Generally, phytoplankton with a small cell size are more sensitive to metal toxicity as they have a larger surface area to volume ratio and higher nutrient uptake efficiency [110]. It has been reported that cyanobacteria are most sensitive to Cu and Cd toxicity, diatoms are the least sensitive, and coccolithophores and dinoflagellates are intermediate in sensitivity [42]. Indeed, a significant decline in *Prochlorococcus* in response to East Asian aerosol amendment has been observed in the oligotrophic seawater of the SCS [64,106,112]. In the coastal regions of the SCS and Yellow Sea, negative responses to aerosol or rainwater addition have also observed in *Synechococcus* and pico-eukaryotes [102,105,106]. It has been reported that the intracellular trace metal concentrations in size-fractionated plankton of the surface water of the NWPO have been significantly elevated relative to their biological requirements due to anthropogenic aerosol deposition [113]. The stronger toxic effect on small phytoplankton may also contribute to the phytoplankton size structure shift to larger phytoplankton. 

Combined metal-to-metal and metal-to-nutrient interactions further complicate the effects of aerosols. For example, Cu toxicity in phytoplankton may be influenced by other metals (e.g., Fe) and nutrient status [111]. In the ECS, phytoplankton growth was more inhibited after the addition of aerosol with high Cu than that with both high Cu and Fe [114]. Coastal strains of some phytoplankton, i.e., *Synechococcus*, exhibit higher Cu tolerance and lower stress response than open-ocean strains [115]. Moreover, although the final yield and growth rate of cyanobacteria decreases in response to aerosol amendment, their cell size and chlorophyll *a* content increases [64,112], which may be due to an uncoupling between photosynthesis and cell division [116]. 

The negative effect of East Asian haze particles on total phytoplankton biomass has only been observed at the very high deposition loadings of 2 mg L^−1^ [103], when the inhibition impact exceeded the fertilization effect, while a stimulation effect was always reported at low and medium loadings of 0.03-0.6 mg L^−1^ [64,103]. Considering that realistic loadings of haze particles is far less than 2 mg L^−1^, the overall effect of atmospheric deposition on phytoplankton biomass should be promotion.

## 6. Effect of East Asian Aerosol on Bacteria

Recent studies on aerosol impacts have begun to focus on the responses of heterotrophic bacteria following aerosol additions. In the oligotrophic ocean, bacterial biomass and production are often limited by dissolved organic carbon. The shortage of inorganic nutrients will also affect bacterial growth directly or indirectly by limiting phytoplankton growth [117]. Therefore, the supply of nutrients and organic matter transported by atmospheric deposition can alleviate the nutritional limitation of bacteria and affect the bacterial activity and diversity. Saharan dust deposition in the Mediterranean Sea and Atlantic Ocean has been shown by microcosm or mesocosm experiments to affect bacterial abundance, production, and community composition [71,103,118,119,120,121,122,123,124]. Far fewer studies have been conducted in the NW Pacific region with East Asian aerosol deposition. 

In the SCS, small or insignificant increases in bacterial abundance in response to anthropogenic East Asian aerosol (collected from Hong Kong and Qingdao) input were demonstrated by microcosm experiments [112,125], whereas significant increases were observed after the addition of dust particles (collected from Mt. Tateyama and Loess Plateau) [126,127]. Bacterial production was enhanced by ~2 to 4-fold, although the increase in bacterial biomass was much smaller [112]. Greater responses in bacterial production than in bacterial abundance have also been reported from the central Atlantic and Mediterranean Sea [118,121]. It is probable that enhanced grazing pressure and viral infection after aerosol addition contribute to maintain a constant bacterial abundance (Figure 1) [102,112,124,128]. Therefore, it has been suggested that the atmospheric input may change the microbial ecosystem from a bottom-up limited to a top-down controlled bacterial community [129]. Alternatively, a shift in bacterial community composition towards one with more active bacteria with higher nucleic acid content after dust addition may also be closely associated with the enhancement of bacterial production [118]. 

Clear changes in bacterial diversity and community composition in NWPO were also detected in response to East Asian aerosol input, although the detailed changes following aerosol additions were site-specific. Generally, the relative abundance of copiotrophs, such as *Rhodobacteraceae* and *Flavobacteriaceae*, increased, while the proportion of oligotrophs, such as *SAR 11* clade, *Prochlorococcus*, *AEGEAN-169* marine group, decreased, leading to a slight increase in bacterial diversity in the oligotrophic SCS [112]. Both bacterial production and the community composition shift exhibited significant relationships with the hydrographic conditions of the different locations. Stronger promotion effects of the East Asian aerosols on bacterial production and community shift from oligotrophs to copiotrophs were demonstrated at the more oligotrophic sites with lower chlorophyll *a* concentration [112]. 

## 7. Future Perspectives

Given that the atmospheric deposition clearly has significant impacts on ocean biogeochemistry, it is important to consider how future alterations to aerosol inputs might influence ocean productivity and the carbon cycle in the NWPO.

(1) Improved modeling of future trends in atmospheric deposition and human impacts, and a better understanding of the responses of marine microbial ecosystems to perturbations by atmospheric inputs are required. More detailed studies of the atmospheric chemistry of EA aerosol deposition and the response of the microbial community, including microzooplankton, phytoplankton, bacteria and viruses, both in short and long terms, are needed, particularly as oceanic and atmospheric records of sufficient length to investigate long-term changes are limited. Thus, time series observations in key regions need to be maintained.
(2) Except for the LNLC regions primarily limited by N, and the HNLC areas significantly affected by Fe, our understanding of the importance and the mechanisms of atmospheric deposition in many areas is still not clear. This is mainly due to uncertainties in the bioavailability and specific composition of atmospheric deposition, and the complexity of the “supply” and “demand” between atmospheric deposition and ocean biota. Moreover, the impact of aerosol deposition on the microbial community is closely related to the nutrient stoichiometry of atmospheric input, initial microbial assemblage, metabolic and trophic state, and the hydrological condition of the investigated water. Therefore, how to quantify the similarities and differences in the responses of marine biota to atmospheric deposition from different sources in different areas and how to determine the controlling factors have become the keys to understanding the impacts of atmospheric deposition on marine primary production processes.
(3) More research on biological mechanisms should be carried out in the future. For example, the detailed mechanisms of the plankton responses on community, individual, and molecular levels and how they influence C, N, P, and S cycles; and the dynamics of microbial food webs, including the bottom-up and top-down effects in response to aerosol input. Moreover, in addition to atmospheric N, P, and Fe, the effects of other trace metals and organic compounds should also be extensively studied. Considering the long residential time and complex chemical and biological interactions of metals and organic matters in the seawater, it is necessary to conduct more experiments to directly assess their impact and combined effect with nutrients on microbes.
(4) As the climate of East Asia is affected by anthropogenic aerosols [130], the combined effects of atmospheric deposition with other environmental changes, such as warming and acidification, should be considered. It has been suggested that predicted warming and acidification will intensify these responses [129], affecting food web processes and biogeochemical cycles.

Although we have established the importance of atmospheric deposition on marine production and plankton communities, there is still a long way to go towards a definite understanding of all the details and impacts so it will be possible to better predict future trends in the atmospheric deposition and their effects. 

## Figures and Tables

**Table 1:** Bioassays with atmospheric dry and wet deposition amendment showing changes of phytoplankton biomass and community structure in NWPO and its marginal seas. The response ratios are the maximum quotients of the mean chlorophyll *a* concentration of the aerosol and control groups during incubation.

	Study Area	Stations	Trophic State	Type of Atmospheric Input	Amendment Concentration (Dry Deposition: mg/L; Wet Deposition: mL/L)	Response Ratio of Chl *a* Concentration	Beneficial Phytoplankton Taxa	Reference
**Dry deposition**	SCS	A3, A6	mesotrophic	Dust (Qingdao, TSP)	1.09	↑2.1–2.9	↑*Chaetoceros* spp.	[85]
		WG2	oligotrophic	Dust (Qingdao, TSP)	1.09	↑3	↑*Nitzschia* spp.	
	SCS	PM7, C3a, A1	mesotrophic	Aerosol (Hong Kong, PM2.5)	0.019 0.19	→ ↑1.7–2.5	Pico- and nano-→ micro-phytoplankton	[64]
		SEATs	oligotrophic	Aerosol (Hong Kong, PM2.5)	0.019 0.19	→ ↑3.5		
	YS	A2	mesotrophic	Dust (Qingdao, TSP)	2 20	→ ↑1.4	Pico-and nano- → micro-phytoplankton	[105]
	ECS	ECS	eutrophic	Mineral dust (Huaniao Island, TSP)	1	↑3	↑Haptophyceae	[104]
				Aerosol (Huaniao Island, TSP)	1	↑2		
				Secondary aerosol (Huaniao Island, TSP)	1	↑1.8	↑Bacillarophyceae, Dinophyceae, Cryptophyceae	
	Kuroshio Extension (KE)	M1	mesotrophic	Dust (Tengger Desert, soil dust)	0.5	↑1.8	Pico- → nano- and micro-phytoplankton	[87]
		M1B	mesotrophic		0.5 1	↑2.5 ↑3		
	Kuroshio-Oyashio transition region (TR)	E10M	eutrophic	Dust (Tengger Desert, soil dust)	0.3 0.5 1	↑1.5 ↑1.7 ↑2.3		
		E2	mesotrophic		0.3 0.5 1	↑2.3 ↑2.8 ↑4.5		
	S-NWPO	Ar4, G7	mesotrophic	Haze particles (Qingdao, TSP)	2	↓0.3–0.8	Pico- → nano- and micro-phytoplankton	[103]
	Kuroshio Extension (KE)	M1B	mesotrophic	Haze particles (Qingdao, TSP)	0.03 0.06	↑2.1 ↑2.6		
		M1	mesotrophic	Haze particles (Qingdao, TSP)	0.1 0.3 0.6	↑1.1 ↑2.0 ↑2.9		
	S-NWPO	A1-b	mesotrophic	Haze particles (Qingdao, TSP)	0.4	↑2.7		
	YS	H10, B7	eutrophic	Haze particles (Qingdao, TSP)	2	↓0.6		
		YS1	mesotrophic	Haze particles (Qingdao, TSP)	0.05 0.1	→		
	ECS	PN3	eutrophic	Haze particles (Qingdao, TSP)	0.4	→		
	S-NWPO	Ar4, G7, K4	mesotrophic	Treated soil dust (Gobi Desert, surface soil)	2	↑1.3–2.8	Pico- → nano- or micro-phytoplankton	[86]
	YS	B7, H10	eutrophic	Treated soil dust (Gobi Desert, surface soil)	2	→		
	S-NWPO	A1-b	mesotrophic	Dust (Mu Us Desert, soil)	0.2 1	↑1.5 ↑2.8	Pico- → nano- and micro-phytoplankton	[88]
	Kuroshio-Oyashio transition region (TR)	B1	mesotrophic	Dust (Mu Us Desert, soil)	0.2 1 2	→ ↑2.0 ↑2.0		
	ECS	C1	eutrophic	Dust (Mu Us Desert, soil)	0.2 1 2	→ ↑1.4 ↑1.6		
	SCS	A7, B2	eutrophic	Aerosols (Guangzhou, TSP)	3.3	↑1.2–1.9	Pico- → micro-phytoplankton	[106]
		C4, A13, B6 C8	mesotrophic	Aerosols (Guangzhou, TSP)	3.3	↑1.2–2.5 ↓0.3		
**Wet deposition**	SCS	A3, A6	Mesotrophic	Rainwater (SCS)	0.4	→		[85]
		WG2	oligotrophic	Rainwater (SCS)	0.4	↑3		
	SCS	R	mesotrophic	Rainwater (SCS R)	50 100	↑1.7 ↑1.9	Pico- → micro- phytoplankton	[107]
				0.7 μm filtered Rainwater (SCS R)	100	↑1.6		
	YS	A2	mesotrophic	Rainwater (SYS)	2	↑1.9	↓Nano-phytoplanton	[105]
	SCS	A7, B2	mesotrophic	Rainwater (Shanwei)	100	↑2–2.5	Pico- → nano- and micro-phytoplankton	[106]
		C4 C8	mesotrophic	Rainwater (Shanwei)	100	↑2.5 ↓0.3		

SCS: South China Sea; ECS: East China Sea; YS: Yellow Sea. ↑ increase in response ratio; ↓ decrease in response ratio; → no significant change.

## Data Availability

Data supporting this study are available in cited articles where they were originally reported.
